# Total soil nutrients drive the enhancement of ecosystem multifunctionality as the succession progresses of the poplar-birch secondary forest

**DOI:** 10.3389/fpls.2025.1708632

**Published:** 2026-01-15

**Authors:** Dongxu Ma, Jiaying He, Qiang Liu, Zhidong Zhang, Lihua Fu, Yue Pang, Jing Tian, Deshuo Kong

**Affiliations:** 1 College of Forestry, Hebei Agricultural University, Baoding, China; 2 Saihanba Mechanized Forest Farm of Hebei Province, Chengde, China

**Keywords:** forest succession, ecosystem multifunctionality, function indices, space-for-time substitution, driving factors, secondary forests

## Abstract

Ecosystem multifunctionality(EMF) refers to an integrated measure of an ecosystem's capacity to perform multiple co-occurring functions. However, change the multi-factor driving mechanism of EMF during poplar-birch secondary forest succession are still poorly understood. Using a space-for-time substitution approach, this study examined four succession stages (early, middle, middle-late, and late) of poplar-birch secondary forests in the Northern Hebei Mountains. It investigated soil physicochemical properties, plant productivity, quantified functional indices and explored the multi-factor driving mechanisms for changing EMF. The results showed that stand and litter (stand volume, litter biomass, litter carbon stock), soil nutrients (organic matter, total nitrogen, available nitrogen, available phosphorus) and soil enzymes (cellobiohydrolase, dissolved organic carbon, n-acetyl-β-D-glucosaminidase and leucine aminopeptidase) significantly elevated as the succession progressed (p < 0.05). Compared with the early stage, the carbon, nitrogen, and phosphorus function indices and the ecosystem multifunctionality index significantly increased by 169%, 287%, 210% and 216% (p < 0.05), respectively. Structural equation modeling (SEM) indicated that increased litter biomass enhanced total soil nutrients, which in turn stimulated soil enzyme activity, ultimately promoting EMF as succession advanced. Notably, total soil nutrients were key factors driving ecosystem multifunctionality enhancement. Overall, plant productivity and soil fertility increased during secondary forest succession, thereby strengthening ecosystem multifunctionality, which provided scientific support for the sustainable development of forest EMF.

## 1 Introduction

Ecosystem functions form the foundation of ecosystem structural stability, primarily involving biogeochemical cycling and ecological processes (Hector and Bagchi, 2007; Smith et al., 2015; Yan et al., 2023). As the dominant component of terrestrial ecosystems, forests play a crucial role in maintaining the energy flow, material cycling, and information transfer (Attiwill and Adams, 1993; Feng et al., 2025). For instance, forests sequester carbon and release oxygen through photosynthesis while providing habitats that sustain biodiversity. Additionally, plants assimilate nitrogen from the soil and synthesize organic compounds, which are subsequently consumed by herbivores and transferred across trophic levels, thereby sustaining biogeochemical cycling and energy flowing ecosystem functionality (Rastogi et al., 2022; Jimenez et al., 2023). Moreover, soil enzymes play a critical role in EMF. On the one hand, it catalyzes the decomposition of organic matter to provide essential nutrients for the forest. Meanwhile, its activity changes are highly coupled with the succession stage (Xue et al., 2022); On the other hand, as an important participant in soil biological activities, promoting nutrient transformation and ensuring the flow and utilization of nutrients (Liu et al., 2023; Neemisha and Sharma, 2022). Existing studies on various ecosystem functions have deepened human theoretical understanding of forest ecosystem single functions. However, forest ecosystem functions are collaborative interactions of multiple components; research on forest ecosystem functions from the perspective of EMF remains scarce. Therefore, investigating forest ecosystem multifunctionality is essential to advance a holistic understanding of forest ecosystem functions.

Ecosystem multifunctionality represents the synergistic performance of multiple ecosystem functions, providing a more comprehensive understanding of ecosystem management (Garland et al., 2021; Lan et al., 2023). The ecosystem multifunctional index is commonly used to quantify ecosystems' capacity to provide and maintain multiple ecological functions, and several studies based on this theoretical parameter have been conducted across diverse ecosystems. For example, examined EMF variations mediated by plants and microorganisms in grasslands using EMF indices; exploring the multifunctionality level of urbanized coastal ecosystems by using the marine EMF index.; and quantified ecosystem functionality in urban green spaces using multifunctionality metrics (Archana and Baker, 2020; Wang et al., 2021b; Cardou et al., 2022). Currently, research on EMF indices across different ecosystems has advanced considerably, offering valuable insights into ecosystem functioning. However, applications of EMF indices in assessing changes induced by forest succession remain limited. Therefore, using this approach to evaluate forest succession dynamics is of great significance for maintaining the sustainability of forest ecosystems.

Forest succession, an important ecological process involving species replacement, resource redistribution, and ecological niche optimization, profoundly influences species composition, biodiversity, soil physicochemical properties, and ecosystem functions (Swanson et al., 2011; Lindenmayer et al., 2019; Han et al., 2023). However, the forest succession process may be altered by various factors. For example, post−fire conditions hinder tree species regeneration and forest succession toward a simpler and less stable state (Skre, 2020). Therefore, in cases of retrogressive succession, artificial interventions are typically implemented to facilitate forest recovery and enhance ecosystem functionality. For instance, artificial interventions can improve light and ventilation conditions in forests, promote the growth of understory vegetation, and increase biodiversity (Romell et al., 2009; Deng et al., 2023). However, human activities act as a double-edged sword; excessive development can disrupt soil ecological balance, posing serious threats to soil health and ecosystem functions (Du et al., 2024; Qu et al., 2024; Mgelwa et al., 2025). Therefore, appropriate artificial intervention is needed, which can not only promote the progress of succession but also not damage ecological health. Additionally, current research on EMF primarily focuses on specific successional stages, while studies on dynamic changes throughout the entire successional process remain relatively scarce. Thus, investigating the response mechanisms of EMF throughout forest succession thus provides a more comprehensive understanding of the temporal dynamics of ecosystem multifunctionality.

Secondary forests, as the main component of forest resources in China, serve as a critical foundation for the sustainable development of forest ecosystem. The northern Hebei mountains bear the ecological protection function of the Beijing-Tianjin-Hebei region, and the poplar-birch secondary forest is one of the main forest types in this area. Poplar-birch secondary forests perform critical ecological functions in the present region, including water conservation, air purification, and biodiversity maintenance (Zeng et al., 2019; Zhang et al., 2025). However, poplar-birch secondary forests have long suffered from anthropogenic damage, resulting in reduced productivity, hindered successional renewal, and constrained ecosystem functions, which collectively fail to meet the region’s ecological requirements (Noormets et al., 2015; Forrester and Bauhus, 2016; Feng et al, 2025). Therefore, in order to enhance the quality of poplar-birch secondary forests, integrated silvicultural measures should be implemented based on the natural succession dynamics of local secondary forest communities (Esteban Lucas-Borja and Delgado-Baquerizo., 2019). Current research on secondary forest has primarily focused on restoring soil fertility, improving forest structure, and enhancing forest stability (Gao et al., 2017; Wang et al., 2022). For example, appropriate artificial intervention can significantly alleviate intraspecific competition and promote tree growth (Fichtner et al., 2012; Condés et al., 2023). Such interventions also reduce tree height-diameter ratios, thereby improving forest mechanical stability, and enhance understory vegetation diversity (Marchi et al., 2018; Yang et al., 2023). Previous studies have deepened our understanding of how artificial intervention promotes the functional development in secondary forests, but the dynamics of EMF during post-intervention succession remain poorly understood. Therefore, investigating the dynamic characteristics of EMF in secondary forests across post-intervention different successional stages can provide a scientific basis for enhancing regional ecological protection functions.

In this study, we selected stands from different poplar-birch secondary forests as the succession progressed in the Northern Hebei Mountains and classified them into four successional stages (early, middle, middle-late, late). We aim to analyze changes in soil physicochemical properties, functional cycling, and ecosystem multifunctionality during the succession process, and to explore the relationship between ecosystem multifunctionality and environmental factors. We hypothesized that (1) soil fertility would improve as succession advanced. As forest composition is optimized and litter quantity increases, soil physicochemical properties improve as the succession progressed; (2) Both single-function indices and the ecosystem multifunctionality index would increase as succession advances. As a result of soil nutrient increases and improvements in single-function indices, the ecosystem multifunctionality index rises as the succession progressed; (3) Ecosystem multifunctionality is co-driven by multiple factors. This may alter multiple ecological factors with the progression of succession that collectively influence ecosystem multifunctionality. By exploring the response mechanisms of ecosystem multifunctionality to secondary forest succession, this study aims to provide insights for enhancing secondary forest quality and promote sustainable development.

## 2 Materials and methods

### 2.1 Study area

The study area is located in the Mulan Paddock (116°32′-117°14′ E, 41°35′-42°40′N) in the western part of Mulan Paddock, Weichang Manchu-Mongolian Autonomous County, Hebei Province, China. The region is characterized by an East Asian mid-temperate semi-humid continental monsoon climate. A frost-free period of 67–128 days is observed, alongside an annual average temperature of 3.3°C and temperature extremes ranging from -42.9°C to 39.4°C. Annual precipitation of 380–560 mm is concentrated mainly in July–September. The predominant soil type is sandy loam (**Supplementary Figure S1**). The main tree species include *Larix principis-rupprechtii*, *Quercus mongolica Fisch. ex Ledeb*, *Betula platyphylla*, *Populus davidiana* and others. The main shrub species are *Vitex negundo* var. *heterophylla, Lespedeza bicolor* and *Spiraea salicifolia*. The main herbaceous species are *Leymus chinensis*, *Taraxacum mongolicum*, and *Sanguisorba officinalis.*

### 2.2 Experimental design

This study employed the " space-for-time substitution " method and took poplar-birch secondary forests at different successional stages as the research objects. By selecting secondary forests with similar site conditions, four different successional stages were determined: early succession stage (E), middle succession stage (M), mid-late succession stage (ML), and late succession stage (L). Specifically, the early succession stage (E) was dominated by pure broad-leaved forest; while the middle succession stage (M), middle-late succession stage (ML), and late succession stage (L) were all *Larix principis-rupprechtii* and *Betula platyphylla* mixed forests (**Supplementary Figures S2**, **Supplementary Figure S3**). The transformation from *Betula platyphylla* secondary forest to·*Larix principis-rupprechtii* mixed forest was achieved through cutting and replanting measures. Three replicate plots were set for each successional stage, with a total of 12 plots. Each plot was 20 m × 20 m in size. The plot spacing was at least 100 m to reduce edge effects.

### 2.3 Sample sampling and indicator measurement

Tree height (H) and diameter at breast height (DBH ≥ 5 cm, measured at 1.3 m) were recorded at each sample plot during the 2023 growing season (July-August). The plants within the sample plots were surveyed to assess community composition. By calculating the volume of wood per tree, the total storage volume for the sample plots could be estimated (For details, please refer to the Materials and Methods section in the supplementary materials). To collect litter, five 1 m × 1 m sample plots were established within each plot to ensure that similar types of wilted material were aggregated. In total, 12 sample plots were utilized, excluding detritus. The collected material was dried at 65°C until a constant mass was achieved, and then weighed. In forest ecosystems, the 0–20 cm topsoil layer, which contains the highest densities of roots, soil microbes, and fauna, serves as the most active hotspot for key processes including organic matter decomposition, nutrient cycling, and enzymatic activities (Fierer et al., 2003; Bardgett and van der Putten, 2014). Therefore soil samples were collected using a soil auger in the vertical direction at depths of 0–20 cm from nine randomly selected "S"-shaped sampling points. Concurrently, the soil was sieved *in situ* with a 2-mm sieve, homogenized, and combined into a single soil sample after removing roots and gravel. Three soil samples were collected from each successional stage, resulting in a total of 12 samples (4 successional stages × 3 samples). The soil samples were then divided into two subsamples. One subsample was air-dried and passed through 0.25 mm and 1 mm sieves for the determination of soil physicochemical properties. The other subsample was frozen in the field and subsequently stored in a laboratory at 4°C for analysis.

Litter carbon stocks were determined by drying to constant weight, pulverizing the litter material, and oxidizing it using potassium dichromate. In addition, soil physicochemical parameters were determined following the guidelines outlined in “Agrochemical Analysis of Soils” (Bao, 2000). The organic matter (OM) content was determined *via* the scorching method. Total nitrogen (TN) was measured using the semi-micro Kjeldahl method. Total phosphorus (TP) was determined by HClO_4_-H_2_SO_4_ digestion and the molybdenum antimony colorimetric method. Total potassium (TK) was determined by the flame photometer method. Dissolved organic carbon (DOC) was determined by the potassium dichromate oxidation method. Available nitrogen (AN) in soil was determined by the indophenol blue colorimetric method. Available phosphorus (AP) was determined by the 0.5 mol/L NaHCO_3_ leaching - molybdenum antimony resist colorimetric method. Available potassium (AK) was determined by ammonium acetate leaching - flame photometric method.

Cellobiohydrolase (CBH), n-acetyl-β-D-glucosaminidase (NAG), leucine aminopeptidase (LAP), and acid phosphatase (AKP) were determined by enzyme activities using the method of Saiya-Cork et al (2002). Weigh 1 g of fresh soil that has passed through a 2-mm sieve, place it in 125 ml of distilled water, and shake it for 2 hours at 25°C and 180 revolutions per minute to prepare a suspension. The sample suspension, substrate solution, and buffer were injected into a 96-well enzyme standard plate in a specific order using a multichannel pipette. After incubation at 25°C for 4 h under light-avoiding conditions, 50 μl of 0.5 mol/L NaOH solution was added to each well to terminate the reaction, and 250 μl was transferred to a labeled 96-well plate (excitation wavelength 365 nm, emission wavelength 450 nm). Soil enzyme activities were calculated after negative control and quenching correction and expressed in units of nmol h¹ g¹.

### 2.4 Data calculation and analysis

Each ecosystem function indicator within the sample was standardized using the Z-score method (for details, see Equation 1) (Mouillot et al., 2011; Maestre et al., 2012; Bradford et al., 2014; Wagg et al., 2014), which is calculated by subtracting the mean value from the measured value and dividing by the standard deviation, as specified in the formula:

(1) $${\text{Z}}_{\text{ij}}=\frac{\left({\text{X}}_{\text{ij}}-{\text{u}}_{\text{i}}\right)}{{\text{σ}}_{\text{j}}}$$

Where Zij is the Z-score of the j ecosystem function indicator of the i sample; Xij is the measured value of the j ecosystem function indicator of the i sample; ui is the mean value of the i ecosystem function indicator within all the samples; and σj is the standard deviation of the j ecosystem function indicator within all the samples.

Each functional indicator is calculated through Equation 2 to obtain:

(2) $${\text{F}}_{\text{ij}}=\frac{\sum_{\text{j}}^{\text{n}}{\text{Z}}_{\text{ij}}}{\text{n}}$$

Where Fij is the function index of the j ecosystem function indicator of the i sample; n is the number of ecosystem function indicators included in the function.

After standardizing the obtained data through z-scores, the ecosystem multifunctionality index (EMF) based on 15 functional variables was evaluated using the commonly employed mean method in multifunctional analysis (Maestre et al., 2012), with the formula as follows

(3) $$\text{EMF}=\frac{\sum_{\text{i}=1}^{\text{F}}{\text{Z}}_{\text{ij}}}{\text{F}}$$

Where EMF is the ecosystem multifunctionality, F is the number of functional parameters, and Zij is the Z-score of the j ecosystem function indicator in the i sample.

A one-way analysis of variance (ANOVA) was conducted on the data using SPSS version 27.0 (International Business Machines Corp) to analyze and compare the differences in plant productivity, soil physicochemical properties, functional index, and multifunctional index at various stages of succession. The significance of the results was assessed using the Tukey HSD method (p < 0.05). Additionally, linear regression analyses were performed on both the functional index and multifunctional index. Box plots illustrating plant productivity, soil physical and chemical properties, functional index, and multifunctional index were created using Origin 2021 software, along with a linear correlation analysis between the functional index and multifunctional index was plotted. In R4.4.2, we used the psych package to analyze the correlation among plant productivity, soil physical and chemical properties, functional index, and multifunctional index. Heat maps were plotted with the corrplot package (Sang et al., 2022) to show the Pearson correlation coefficients of EMF with soil physicochemical properties, total soil nutrients, soil available nutrients, and enzyme activities. The Boruta algorithm was employed through the survival package in R to identify key factors and generate a feature importance ranking plot (Kursa and Rudnicki, 2010). Using the pls-pm software package (Zhou et al., 2024), we constructed a structural equation model to quantify the direct and indirect effects of succession stage, litter, total soil nutrients, total available soil nutrients, and soil enzymes on EMF, with a goodness-of-fit (GOF) threshold of ≥0.7 for model acceptance. ArcMap 10.8 software was used to map the distribution of the sample plots.

## 3 Results

### 3.1 Changes in plant factors and soil physicochemical properties as the succession progressed

**Figure 1:**
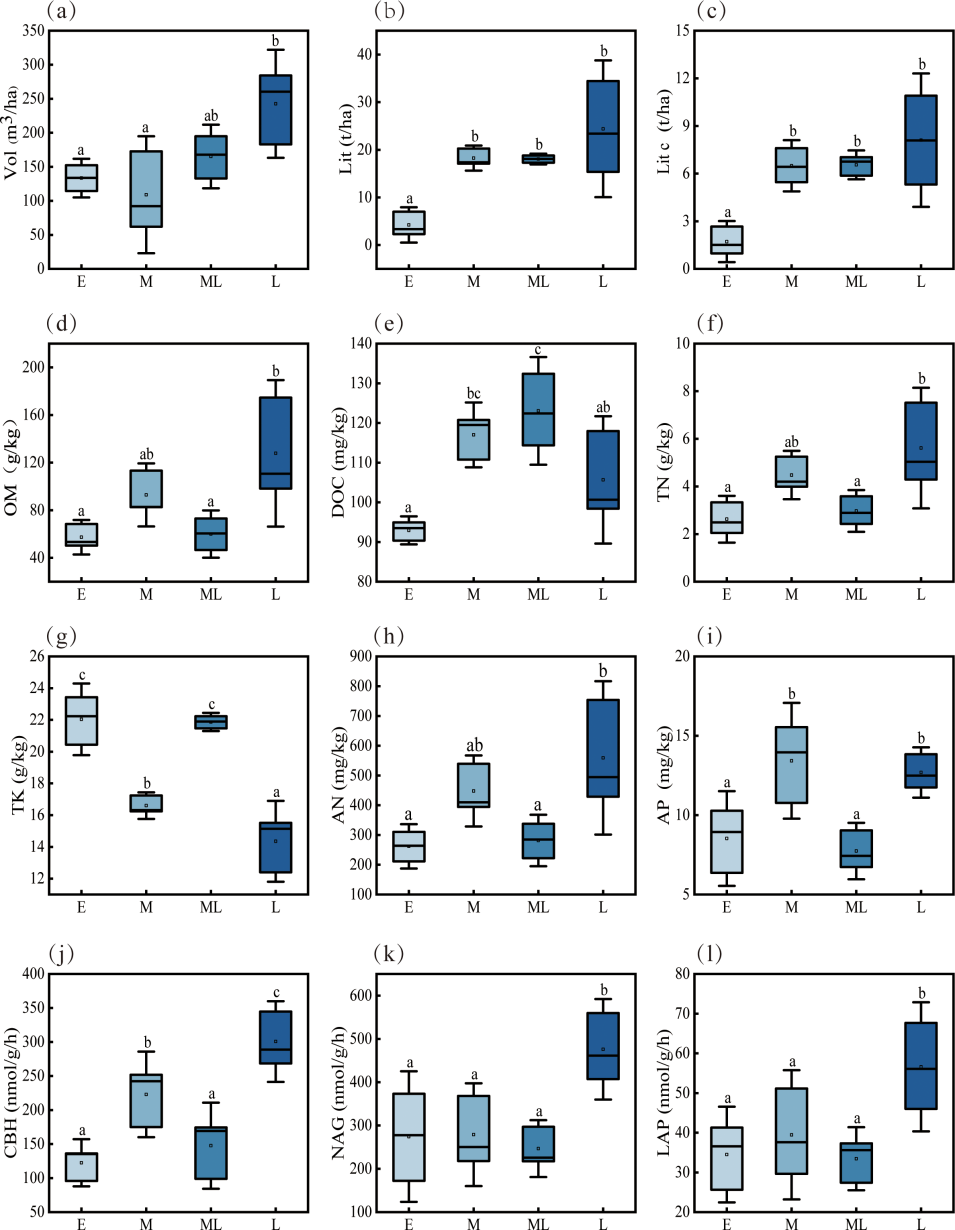
Vegetation and soil factors during the succession process. E, M, ML, and L represent the early succession stage, middle succession stage, mid-late succession stage, and late succession stage, respectively. Vol: stand volume; Lit: litter biomass; lit c: litter carbon stock; OM: organic matter; DOC: dissolved organic carbon; TN: total nitrogen; TK: total potassium; AN: available nitrogen; AP: available phosphorus; CBH: cellobiohydrolase; NAG: n-acetyl-β-D-glucosaminidase; LAP: leucine aminopeptidase.

During forest succession, most plant and soil metrics increased significantly (**Figure 1** p < 0.05). Vol, Lit, and Lit C showed the most pronounced increases (81.8%, 478.6%, and 372%, respectively), followed by OM (122.7%), TN (113.9%), AN (113.4%), and enzyme activities (CBH: 145.5%; NAG: 73.4%; LAP: 64.1%). DOC and AP increased modestly (13.7% and 48.8%).

### 3.2 Changes in single-function and multifunctionality indices and correlation

**Figure 2:**
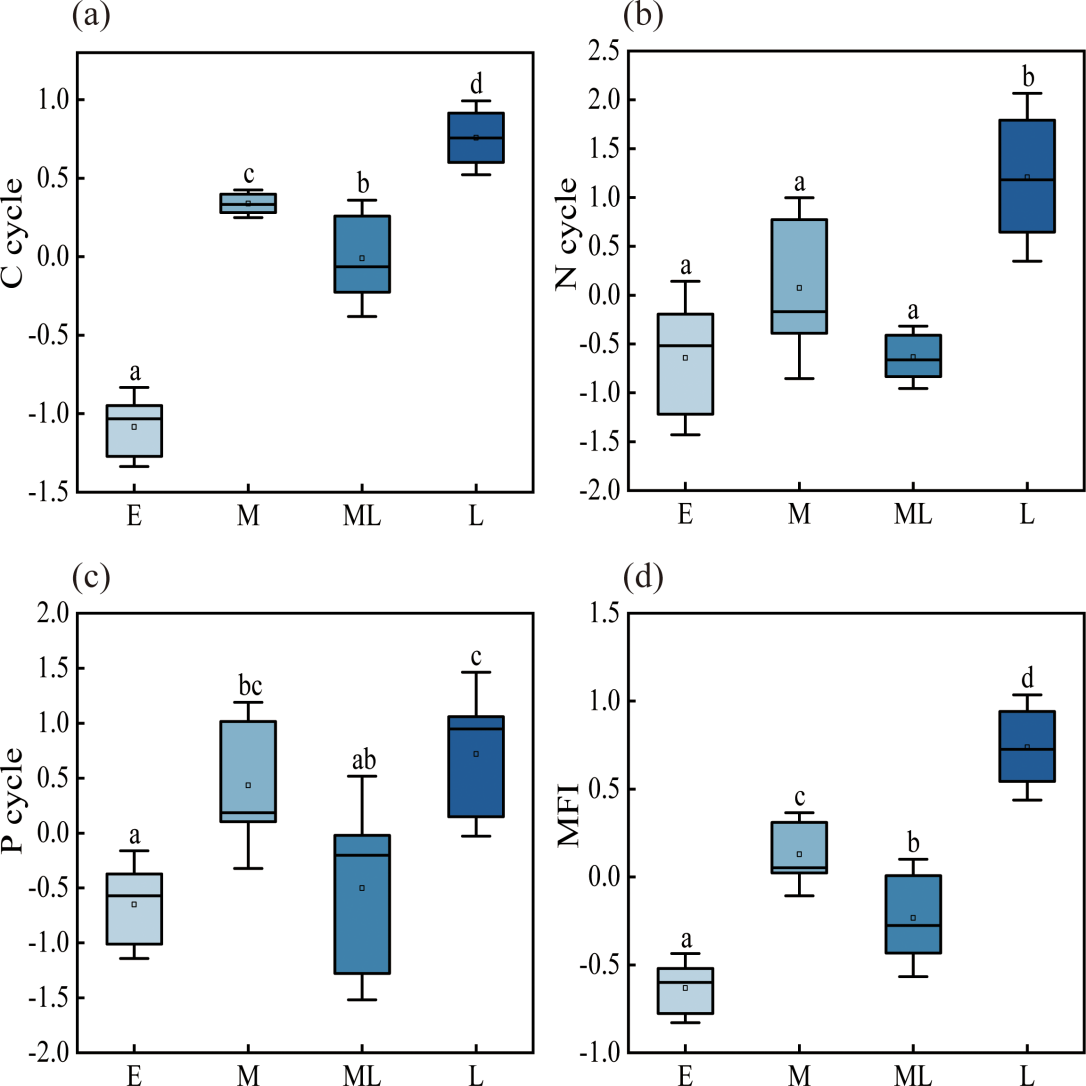
Single-function indices and ecosystem multifunction index as the succession progressed. C cycle: carbon function index; N cycle: nitrogen function index; P cycle: phosphorus function index; MFI: multifunctional index of ecosystems.

As succession progressed, the carbon, nitrogen, and phosphorus function indices increased significantly by 169%, 287%, and 210%, respectively, compared with the early succession stage (**Figures 2a–c**, p < 0.05). In contrast, the potassium cycling index showed no significant change throughout succession (**Supplementary Figure S5**, p > 0.05). The ecosystem multifunctionality index also increased significantly, peaking in the late succession stage with a 216% rise relative to the early stage (**Figure 2d**, p < 0.05).

**Figure 3:**
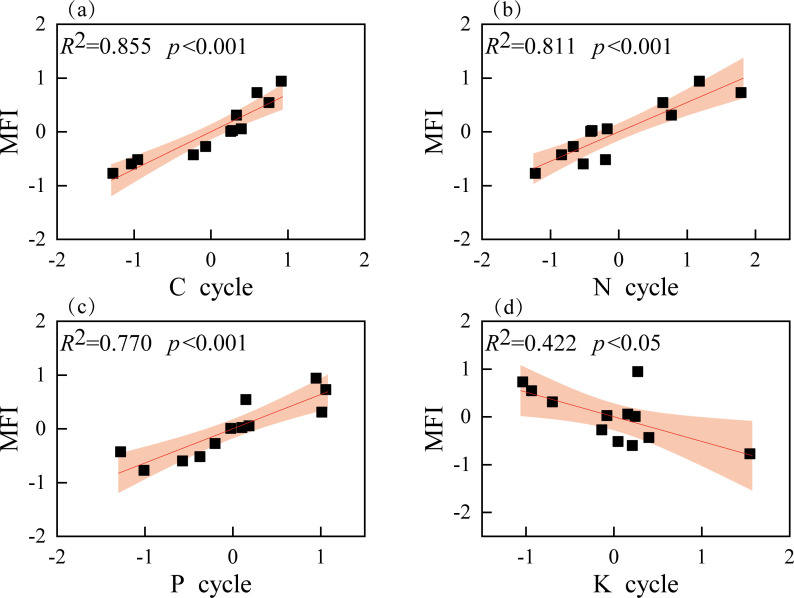
Relationship between single function index and ecosystem multifunctionality index during succession. Shaded areas are 95% confidence intervals of the fit.

Linear regression analysis revealed significant positive correlations between the carbon, nitrogen, and phosphorus cycling indices and ecosystem multifunctionality (**Figure 3a–c**, p < 0.05), whereas the potassium cycling index showed a significant negative correlation (**Figure 3d**, p < 0.05).

### 3.3 Driving mechanisms of ecosystem multifunctionality

**Figure 4:**
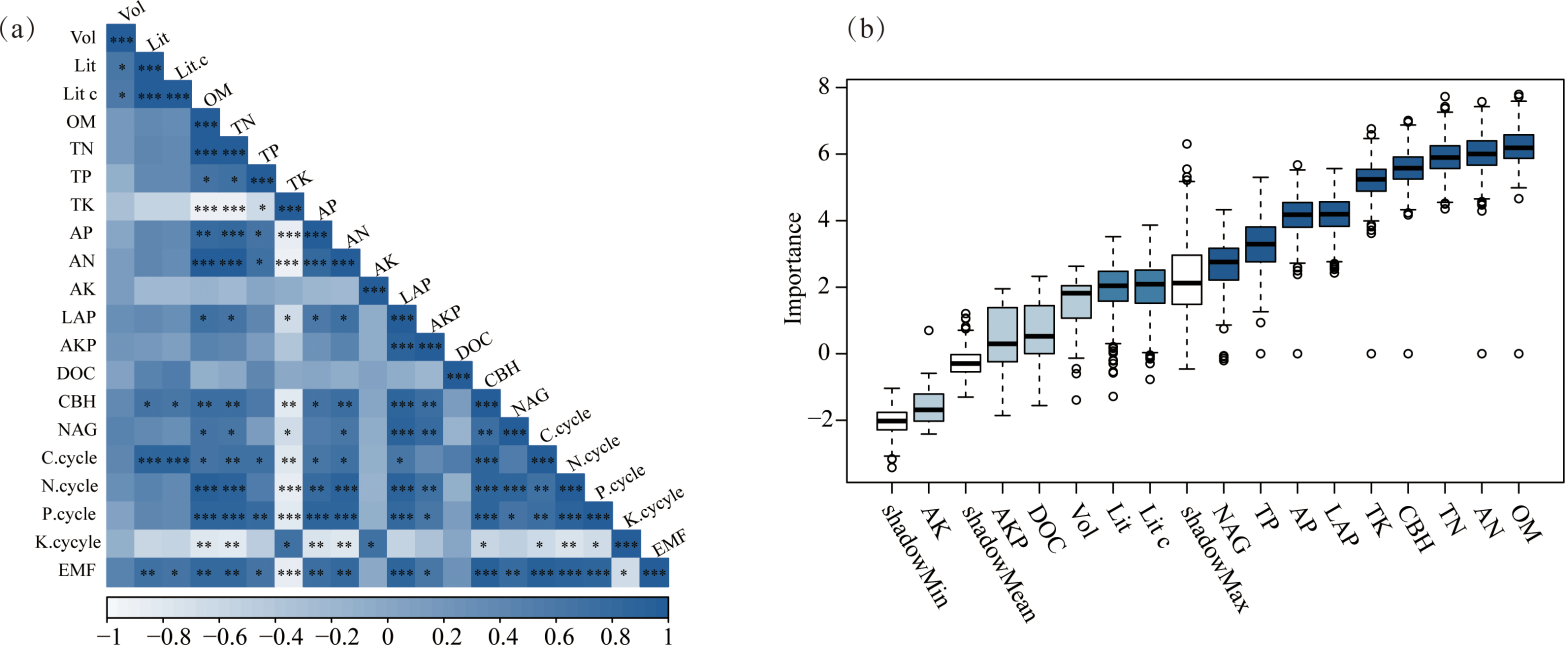
Relationship between EMF and ecological factors. **(a)** Pearson correlation analysis of EMF. **(b)** Selection of significant predictors of factors affecting the ecosystem function index. In the Boruta algorit, significance testing was conducted. The maximum importance of shadow features (max_shadow) was derived through multiple iterations. Variables were deemed "successful" if their importance exceeded max_shadow, and "failed" otherwise. A feature was labeled "confirmed" (significantly correlated) if the number of successes (k) was significantly higher than expected at p=0.5, and "excluded" (no significant correlation) if significantly lower.

EMF was significantly correlated with plant factors and soil physicochemical properties. Pearson’s correlation analysis showed significant positive correlations between EMF and Lit, Lit C, OM, CBH, NAG, TN, AN, LAP, AP, and TP (**Figure 4a**, p < 0.05), but a significant negative correlation with TK (**Figure 4a**, p < 0.05). The Boruta screening identified OM, AN, TN, CBH, TK, LAP, AP, TP, and NAG as relatively important indicators (**Figure 4b**), consistent with the correlation results.

**Figure 5:**
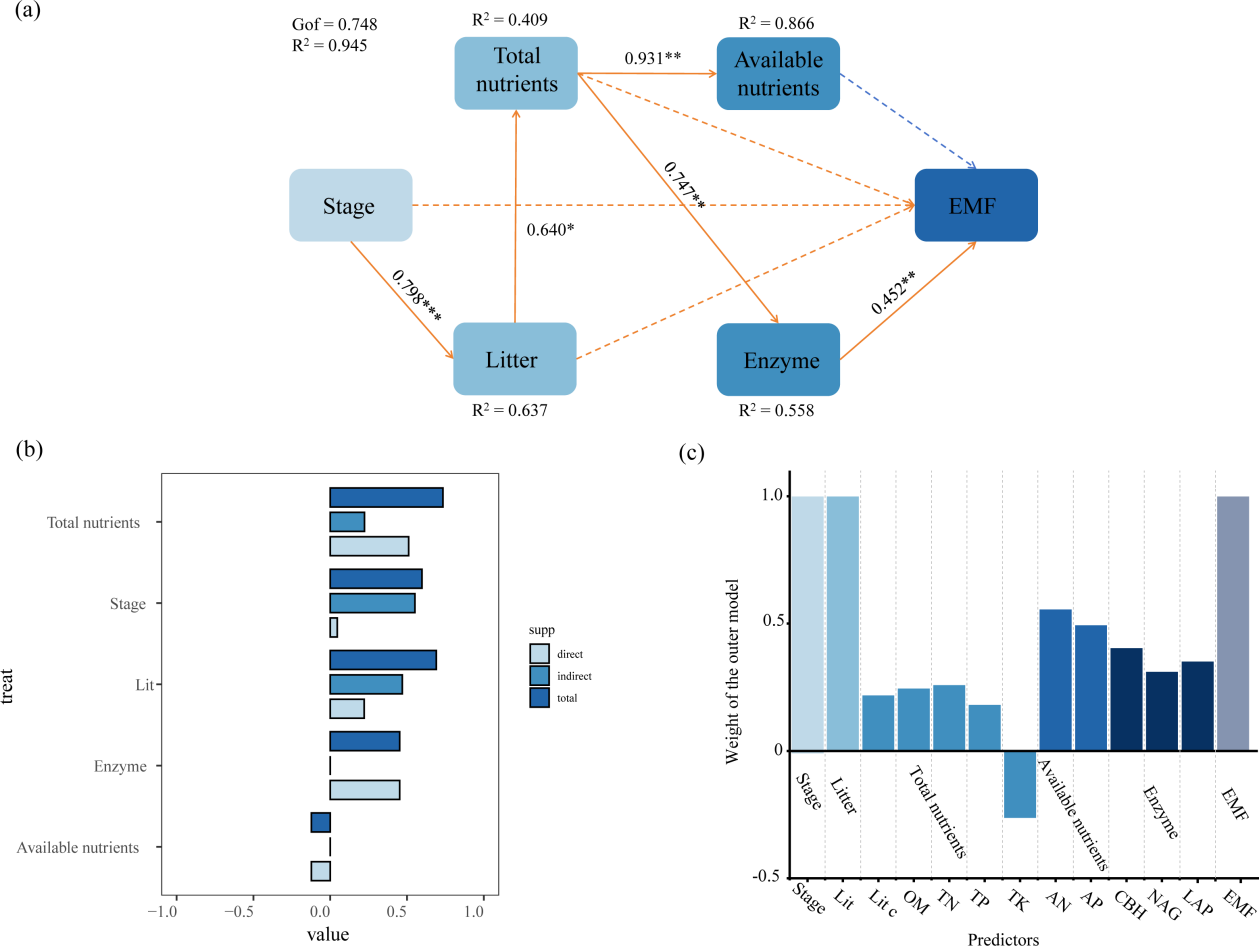
Partial least squares path analysis showing effects of succession on EMF. Structural equation modeling illustrating the causal relationships of litter, total soil nutrients, available nutrients, Enzyme, and EMF **(a)**; Contributions of each direct-indirect and total effect value. Negative on the left side and positive on the right side **(b)**; Weight of the outer model. Upward is positive, downward is negative **(c)**.

PLS-PM analysis further revealed the direct and indirect pathways affecting EMF (**Figure 5**). Litter had a strong positive direct effect on total soil nutrients (0.640). Total soil nutrients positively influenced soil enzymes and available nutrients (0.747 and 0.931, respectively). Soil enzymes exerted a positive direct effect on EMF (0.452). Overall, total soil nutrients exhibited the strongest total positive effect on EMF (**Figure 5b**).

## 4 Discussion

### 4.1 Improvement in plant productivity and soil fertility as the succession progressed

Community species composition shifts during succession progression, and the enhanced resource utilization capacity of dominant species facilitates net primary productivity (NPP) accumulation (Gu et al., 2025). Furthermore, dynamic changes in Vol exhibit a significant positive correlation with long-term variations in NPP. In the present study, the tree species mixing ratio changed as succession advanced (**Supplementary Figure S3**), and the elevated proportion of dominant species promoted rapid Vol accumulation (**Figure 1**). Notably, an increased proportion of dominant species not only drives biomass accumulation, but also strengthens the carbon sequestration capacity of plants (Chen et al., 2018). On one hand, the increases in Lit and Lit c are attributed to enhanced biomass; on the other hand, they result from improved functional traits of dominant species. Thus, the higher proportion of dominant species and optimization of their functional traits drive improvements in plant productivity, thereby enhancing various related indicators. In the present study, Vol, Lit, and Lit c all exhibited significant increases—this observation indicates that plant productivity was enhanced as succession progressed. Numerous studies confirmed that plant community structure gradually became more complex and plant diversity continuously increased as the succession progressed (Liu et al., 2020; Xu et al., 2020). In our study site, the tree species composition shifted from pure broadleaf species to a mixed coniferous-broadleaf forest as the succession moved toward later stages. This transformation not only promoted a more rational stand structure but also increased vegetation species diversity (**Supplementary Figure S3**). Studies indicated that coniferous and broadleaf tree species differed in various aspects, including their nutrient requirements (Bu et al., 2020; Liu et al., 2023). Therefore, in our study, coniferous and broadleaf tree species utilized different nutrients, reducing competitive pressure further promotes the improvement of productivity.

Soil serves as the basis for plant survival, and plants in turn nourish the soil. Both played a crucial role in maintaining sustainable forest development (Jamir et al., 2019; Krüger et al., 2019). We found that OM, TN, AN, and AP all increased significantly as the successional process proceeded, promoting the recovery of soil fertility. Early extensive research showed that litter was not only a significant source of soil nutrients but also key to increase soil fertility (Sauvadet et al., 2020; Gulati and Kaur, 2024). The present work, enhanced plant productivity produced large amounts of litter, supplying more decomposable material and thereby returning nutrients to the soil as the succession progressed. Simultaneously, litter provided soil microorganisms with large amounts of organic compounds, enhancing soil microbial activity (Blesh and Ying, 2020). In the early stages of this study, low litter biomass failed to meet the demands of microbial activity, resulting in low soil nutrient levels in early succession. Therefore, as the succession advanced and litter biomass increased, it met the energy demands of soil microorganisms, decomposition efficiency improved, more nutrients entered the soil, and soil fertility was restored.

We found that CBH, NAG, and LAP all increased significantly as the succession progressed. This was likely because litter contained abundant organic matter, providing substrates for soil enzyme synthesis (Nie et al., 2023). Our results indicate, the increased litter biomass returned more organic matter to the soil, meeting the synthesis material requirements of soil enzymes as the succession transitioned from early to late stages. Furthermore, soil microorganisms produced large amounts of soil enzymes during their metabolic processes (Hemati et al., 2021). In this study, insufficient litter biomass in the early successional stages limited soil microbial metabolism, resulting in low soil enzyme activity. As the succession progressed, increased litter biomass met the demands of soil microbial activity, resulting in the secretion of substantial amounts of soil enzymes.

Additionally, we found significant positive correlations between OM, TN, AN, AP, and soil enzyme activities. Soil enzymes played an important role in nutrient accumulation (Shi, 2011). In contrast, total potassium content decreased as the succession proceeded. On one hand, potassium’s high solubility (Goulding et al., 2021). The soil texture in our study area was mainly sandy loam (**Supplementary Figure S1**), which had good water permeability. Combined with concentrated precipitation from July to September, potassium dissolved in rainwater was leached away, accelerating the decline in soil total potassium content (Wang et al., 2021a). On the other hand, different stand types have varying demands for potassium, and their impact on soil total potassium content may also differ (Das, 2021). As the succession progressed, vegetation continuously renewed, plant growth accelerated, and large amounts of potassium were absorbed, which may have led to a decrease in soil total potassium content. Overall, plant productivity and soil fertility improved from early to late successional stages, supporting our first hypothesis.

### 4.2 Increase in functional indices and ecosystem multifunctionality indices as the succession progressed

Each function played a crucial role in the ecosystem, and ecosystem multifunctionality integrated the synergistic effects of multiple functions (Li et al., 2023; Shinfuku et al., 2024). The single-function indices for carbon, nitrogen, and phosphorus significantly increased, which was consistent with previous research findings, as the succession advanced (Ma et al., 2020). In this study, plant composition was relatively simple in the early succession stages (**Supplementary Figure S3**), and litter biomass was low. This limited litter biomass failed to meet the demands of soil microbial activity, resulting in reduced nutrient input and constrained nutrient uptake and transformation (Miao et al., 2019). Therefore, these function indices were relatively low in the early stages. We found that the carbon, nitrogen, and phosphorus function indices increased as the succession progressed. This was likely because the increase in litter biomass enhanced soil microbial activity, thereby accelerating carbon, nitrogen, and phosphorus cycling (Wei et al., 2020). On the other hand, differences in root ecological niches and varying nutrient requirements between coniferous and broadleaf tree species created a complementary effect, promoting nutrient transformation and cycling (Zhang et al., 2023). In this study, tree species gradually diversified, litter biomass met the demands of soil microbial activity, enhancing microbial activity as the succession transitioned from early to late stages (**Supplementary Figure S3**). This, in turn, promoted the release of nutrients into the soil, improved soil fertility, supplied nutrients to the stand, and formed a nutrient cycle. Simultaneously, soil microorganisms secreted more soil enzymes. These enzymes actively participated in carbon, nitrogen, and phosphorus cycling, and the increase in function indices reflected their positive contribution to nutrient cycling and transformation. In contrast, the single functional index for potassium did not change significantly. This might be because potassium was leached, reducing the amount available for nutrient transformation and cycling, thus preventing significant changes in the functional index (Kayser and Isselstein, 2005; Sindhu et al., 2014). On the other hand, continuous plant renewal may have maintained a high potassium demand as the succession progressed, but nutrient return did not increase. Consequently, less potassium was retained in the soil, further explaining the lack of significant changes in potassium function toward later successional stages.

The ecosystem multifunctionality index exhibited significant positive correlations with the single function indices for carbon, nitrogen, and phosphorus as the succession progressed. Numerous studies have shown that the increasing complexity of vegetation community structure drives the enhancement of functional indices during successional development (Gong et al., 2020; Tu et al., 2022). In our study, the transition from pure broadleaf to mixed coniferous-broadleaf forest promoted diversification of the stand structure and increased plant productivity, generating substantial litter, as the succession progressed. Previous studies have shown that stable nutrient supply supported the diversity and activity of the soil microbial community (Leff et al., 2015; Dai et al., 2019; Qian et al., 2022). In this study, the increase in litter biomass provided soil microorganisms with an abundant carbon source. Meanwhile, the large amount of litter satisfied the requirements of microbial activity, thereby returning substantial nutrients to the soil (Stirling et al., 2019). We observed that soil fertility significantly improved as the successional process proceeded, altering the soil nutrient environment in our study. Numerous studies have shown that improved nutrient availability provides ample nutrition for plant growth, increases photosynthetic efficiency and productivity, and promotes energy flow (Megersa, 2021; Yadav et al., 2021). Our research found that the increase in litter biomass, the enhancement of soil microbial activity, and the improvement in plant nutrient supply formed a positive feedback loop, ultimately collectively enhancing ecosystem multifunctionality. Moreover, in the present study, nutrient cycling occurred from plants to soil and back to plants. We used functional indices and ecosystem multifunctionality indices to further clarify the relationship between ecological factors and ecosystem multifunctionality. Therefore, improvements in various single functions collectively contributed to the enhancement of ecosystem multifunctionality (**Figure 3a-c**, **4**). In summary, the carbon, nitrogen, and phosphorus function indices and the ecosystem multifunctionality index all significantly increased as the succession progressed, supporting the second hypothesis.

### 4.3 Driving mechanisms for the enhancement of ecosystem multifunctionality

Multiple ecological factors changed, thereby affecting ecosystem multifunctionality, as the succession shifted to more mature stages. Previous studies have shown that ecosystem multifunctionality was synergistically influenced by multiple factors, and ecosystem changes were complex processes (Callaghan et al., 2013). Therefore, this study identified key driving factors, facilitating a systematic understanding of the internal mechanisms of ecosystem multifunctionality changes during succession. Previous studies indicated that as the succession progressed, litter decomposed more readily, returning substantial nutrients to the soil, and improved soil nutrients contributed to enhanced ecosystem multifunctionality (Esteban Lucas-Borja and Delgado-Baquerizo, 2019; Liu et al., 2021). The structural equation model (**Figure 5a**) clearly illustrated the pathway through which litter biomass increased total soil nutrients across succession stages, stimulated soil enzyme activity, and consequently enhanced ecosystem multifunctionality. Litter nutrient return was a key pathway for soil nutrient replenishment (Chomel et al., 2016). This investigation established that increased litter biomass returned large amounts of decomposable material to the soil, promoting soil nutrient recovery as the succession advanced. The restoration of soil nutrients stimulated an increase in soil enzyme activity. Existing studies demonstrated that soil nitrogen and phosphorus are essential components of enzymes, and their synthesis requires substantial nitrogen and phosphorus inputs (Kishorekumar et al., 2020; Walia and Kaur, 2024). Our results indicated that TN, AN, and AP increased significantly as succession progressed, providing sufficient substrates and raw materials for soil enzyme synthesis. Furthermore, improved soil nutrients enhanced the soil microbial environment, promoting enzyme synthesis and secretion (Zhang et al., 2019; Wang et al., 2023). We found that increased organic matter content provided abundant energy and nutrients for soil microorganisms as the successional process proceeded. Enhanced microbial activity subsequently led to greater secretion of soil enzymes. In summary, multiple factors such as plants, litter, soil, and microorganisms jointly drive the dynamic evolution of the ecosystem multifunctionality in secondary forests through a complex synergistic network, during ecosystem succession.

The direct and indirect effects diagram (**Figure 5b**) further revealed how various factors influenced ecosystem multifunctionality and their respective contributions. Specifically, different succession stages exerted stronger indirect effects on ecosystem multifunctionality, primarily mediated through increased litter biomass affecting total soil nutrients and soil enzyme activity during succession. Notably, total soil nutrients exhibited the greatest total effect on ecosystem multifunctionality, highlighting their pivotal role in enhancing both ecosystem productivity and multifunctionality. On one hand, the return of substantial amounts of litter to the soil provided a major nutrient supplement (Wang et al., 2024). On the other hand, the organic matter within the litter served as an abundant substrate for soil enzymes; moreover, during its decomposition, litter altered the soil environment, thereby influencing soil microbial activity (Gong et al., 2023). Additionally, in this study, soil enzyme activity exerted a significant positive direct effect on ecosystem multifunctionality. This was attributable to soil enzymes directly participating in nutrient cycling and transformation processes, promoting the accumulation of plant nutrients, improving plant productivity, and ultimately driving the enhancement of ecosystem multifunctionality (Neemisha and Sharma, 2022). Our results validated that soil enzymes facilitated the cycling of carbon, nitrogen, and phosphorus nutrients, promoting synergistic interactions among multiple functions, thereby strengthening ecosystem multifunctionality.

The structural equation modeling indicated that the available nutrients had no significant effect on ecosystem multifunctionality, suggesting that ecosystem multifunctionality might not be regulated by these nutrients as succession advanced. Previous studies presented debates regarding the role of available nutrients. Specifically, complex interactions might exist between available nutrients and soil microbial communities, where microbial activity influences nutrient conversion efficiency, thereby affecting ecosystem functions (Yang et al., 2024). Conversely, the high tannin content of apoplasts and the slow decomposition of cellulose result in prolonged nutrient retention, thereby reducing nutrient availability (Ezekiel et al., 2022). Therefore, the impact of available nutrients on ecosystem multifunctionality remained controversial. In the current study, the lack of a significant effect of available nutrients on ecosystem multifunctionality aligned with the conclusions of the latter perspective. This pattern arose because increased coniferous species produced more high-tannin litter that decomposed slowly, leaving nutrients in the soil, thereby diminishing the impact of available nutrients on ecosystem multifunctionality, as the succession progressed (**Supplementary Figure S3**). Overall, the structural equation model demonstrated that increased litter biomass, enhanced total soil nutrients, and improved soil enzyme activity collectively exerted significant positive effects on ecosystem multifunctionality as the succession advanced. Among these factors, total soil nutrients were identified as the key driver, contributing most substantially to the enhancement of ecosystem multifunctionality. This finding supported the third hypothesis.

## 5 Conclusions

This study examined the dynamics and drivers of ecosystem multifunctionality in poplar and birch secondary forests in western Mulan Paddock, Hebei Province. With forest succession, plant productivity, soil fertility, and functional indices increased, enhancing overall ecosystem multifunctionality. Increased litter biomass promoted soil nutrient recovery and enzyme activity, ultimately improving multifunctionality. Total soil nutrients were identified as the key predictor. These findings provide a theoretical basis for enhancing ecosystem multifunctionality in cold-temperate secondary forests and underscore the need for multidisciplinary approaches to optimize management under global change.

## Data Availability

The raw data supporting the conclusions of this article will be made available by the authors, without undue reservation.
