# Supplementary material for: Total soil nutrients drive the enhancement of ecosystem multifunctionality as the succession progresses of the poplar-birch secondary forest
Source: Front Plant Sci. 2026 Jan 15;16:1708632. doi: 10.3389/fpls.2025.1708632 (PMC12854143; doi:10.3389/fpls.2025.1708632)
Supplement: Supplementary file 1 [file DataSheet1.pdf]

Supplementary Item

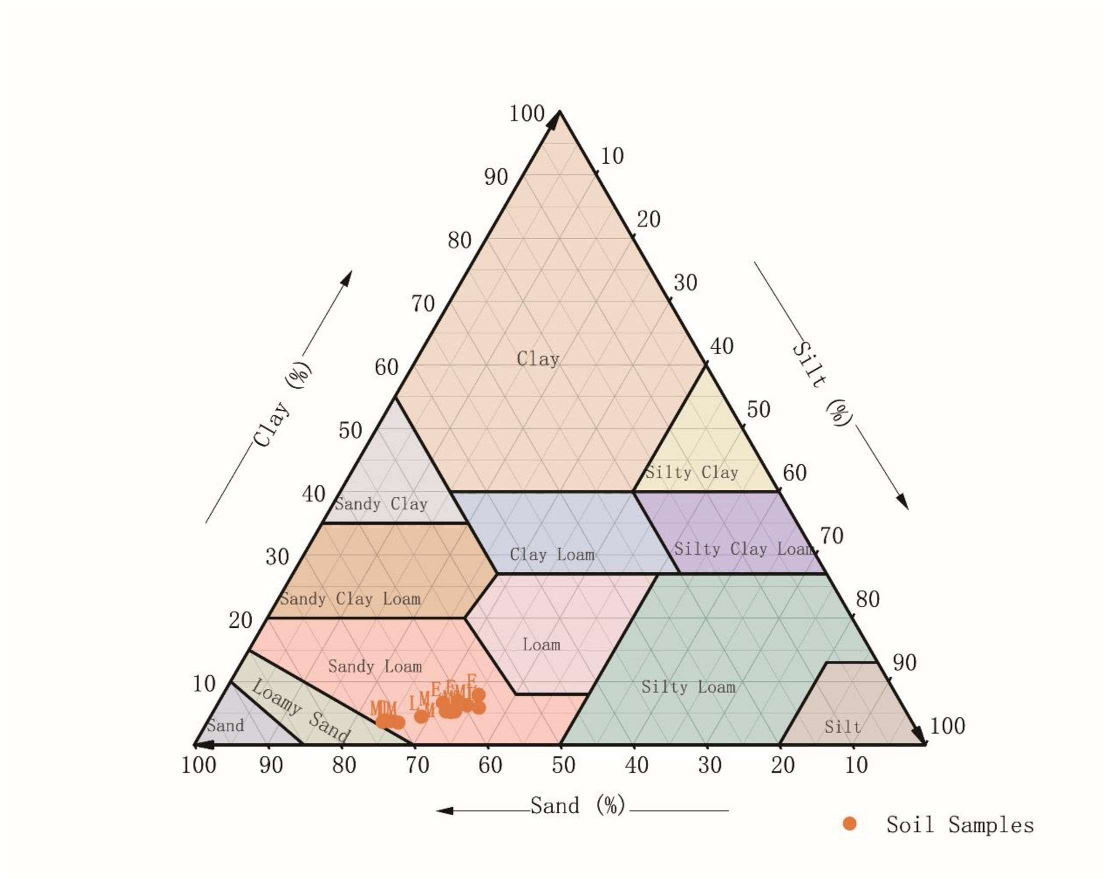

Fig. S1 soil texture structure diagram of the equilateral triangle.

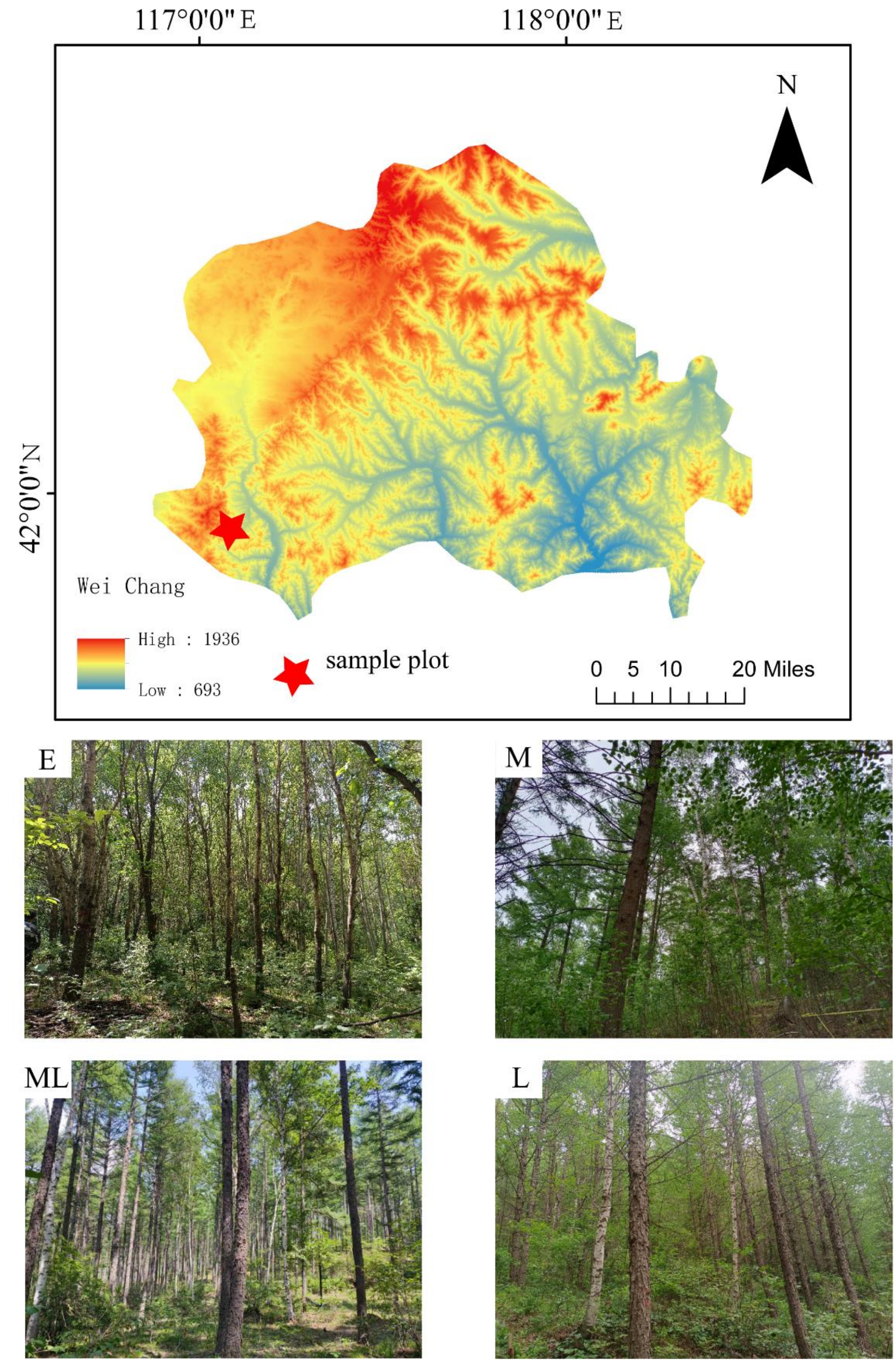

Fig. S2 Location map of the sample area. E M, ML, and L represent the early succession stage, middle succession stage, mid-late succession stage, and late succession stage, respectively. Field images of different successional stages.

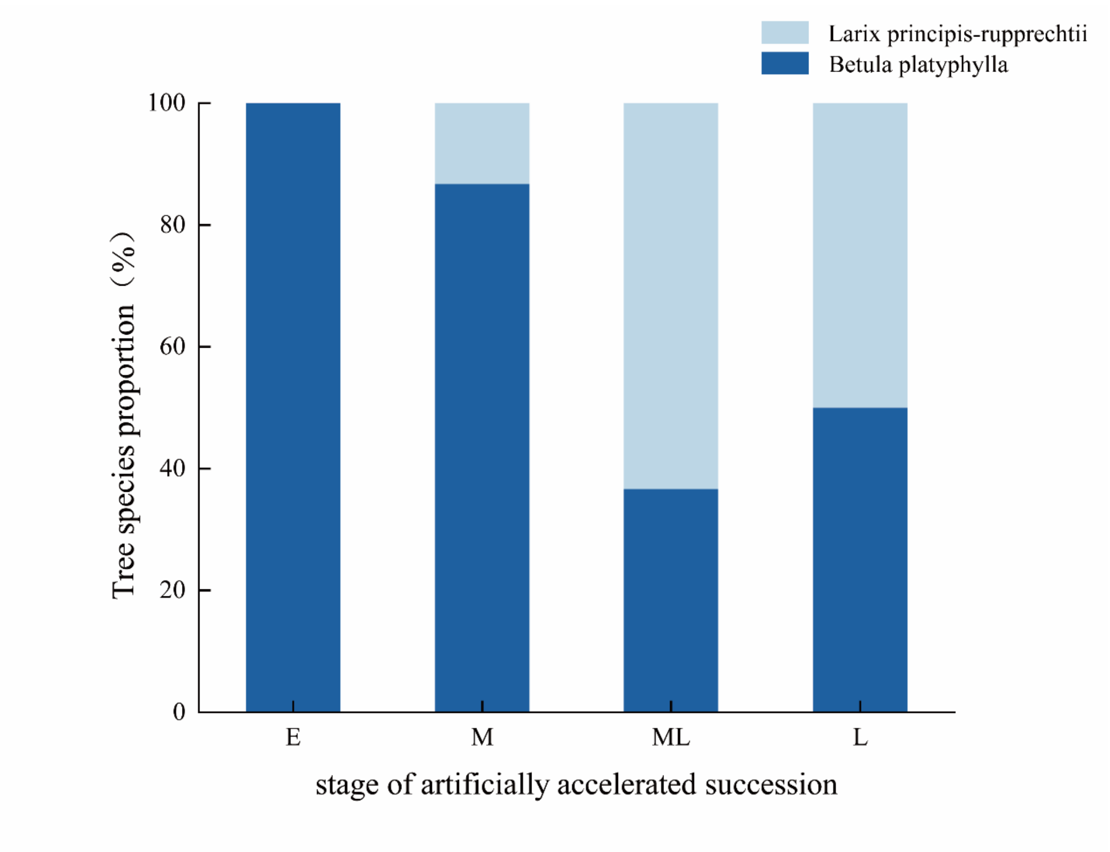

Fig. S3 Changes in tree species proportions during succession. E M, ML, and L represent the early succession stage, middle succession stage, mid-late succession stage, and late succession stage, respectively.

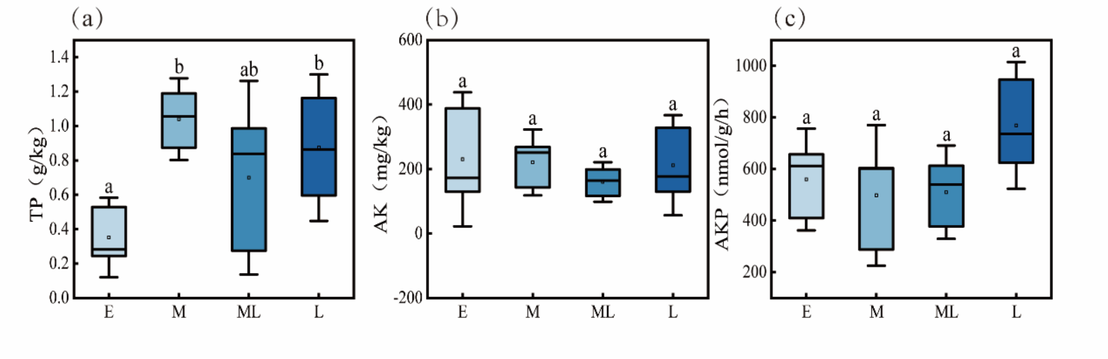

Fig. S4 Soil factors during the succession process. E M, ML, and L represent the early succession stage, middle succession stage, mid-late succession stage, and late succession stage, respectively. TP: total phosphorus; AK: available potassium; AKP: acid phosphatase.

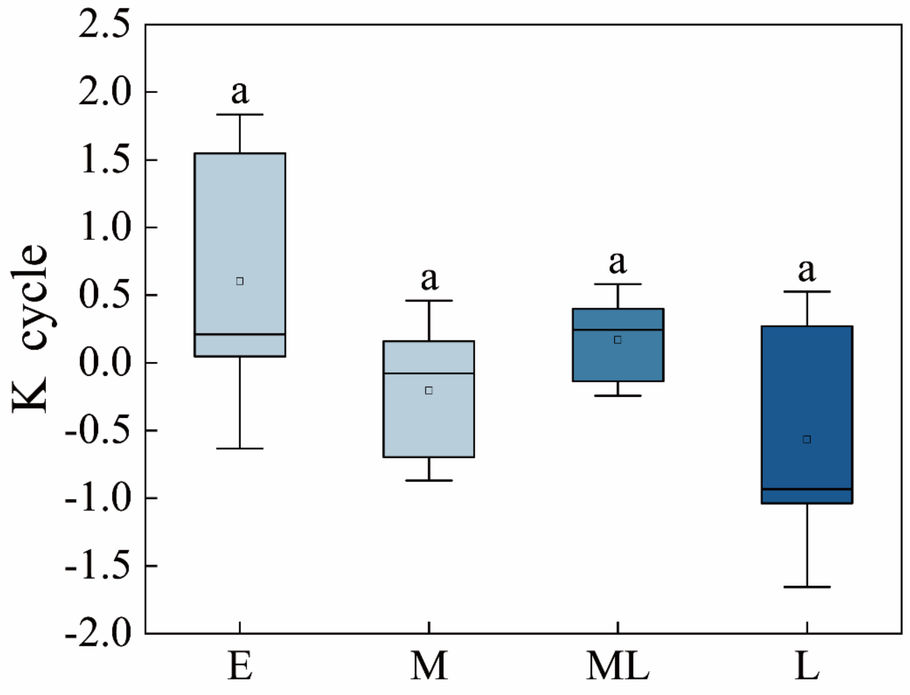

Fig. S5 Single-function indices as the succession progressed. K cycle: Potassium function index.

Materials and Methods

Data calculation

Stand stock volume calculation

For each tree in the sample plot, record the diameter at breast height (DBH), height and other information. Use the univariate volume formula to calculate the volume of each tree, and then estimate the volume of each tree per unit area. The specific formula is as follows:

M=a×DBHb (1)

In the formula, M represents the volume of a single tree, D represents the diameter at breast height (the diameter of the tree trunk at 1.3 meters above the ground, in cm), H represents the tree height (in m), and A and b are model parameters. *Betula platyphylla* model: a=0.16415, b=2.40688; *Larix principis-rupprechtii* model: a=0.09477, b=2.56081

$$Vol=\frac{\sum M_i \times N}{10000} \quad (2)$$

In the formula, Vol stands for standing stock per unit area, $\sum M_i$ is the sum of the volume of each individual tree (M), N represents the number of trees per unit area.
